# Synthesizing 4D Magnetic Resonance Angiography From 3D Time-of-Flight Using Deep Learning: A Feasibility Study

**DOI:** 10.7759/cureus.60803

**Published:** 2024-05-21

**Authors:** Akihiko Wada, Toshiya Akatsu, Yutaka Ikenouchi, Michimasa Suzuki, Toshiaki Akashi, Akifumi Hagiwara, Mitsuo Nishizawa, Katsuhiro Sano, Koji Kamagata, Shigeki Aoki

**Affiliations:** 1 Department of Radiology, Juntendo University School of Medicine, Tokyo, JPN; 2 Department of Radiology, Juntendo University Hospital, Tokyo, JPN; 3 Department of Radiology, Juntendo University Urayasu Hospital, Chiba, JPN

**Keywords:** neuro mri, u-net, lstm networks, deep convolutional network, deep neural network, asl mra, tof mra

## Abstract

Objective and background

This study aimed to develop a deep convolutional neural network (DCNN) model capable of generating synthetic 4D magnetic resonance angiography (MRA) from 3D time-of-flight (TOF) images, allowing estimation of temporal changes in arterial flow. TOF MRA provides static information about arterial structures through maximum intensity projection (MIP) processing, but it does not capture the dynamic information of contrast agent circulation, which is lost during MIP processing. Considering the principles of TOF, it is hypothesized that dynamic information about arterial blood flow is latent within TOF signals. Although arterial spin labeling (ASL) can extract dynamic arterial information, ASL MRA has drawbacks, such as longer imaging times and lower spatial resolution than TOF MRA. This study's primary aim is to extend the utility of TOF MRA by training a machine-learning model on paired TOF and ASL data to extract latent dynamic information from TOF signals.

Methods

A DCNN combining a modified U-Net and a long-short-term memory (LSTM) network was trained on a dataset of 13 subjects (11 men and two women, aged 42-77 years) using paired 3D TOF MRA and 4D ASL MRA images. Subjects had no history of cerebral vessel occlusion or significant stenosis. The dataset was acquired using a 3T MRI system with a 32-channel head coil. Preprocessing involved resampling and intensity normalization of TOF and ASL images, followed by data augmentation and arterial mask generation. The model learned to extract flow information from TOF images and generate 8-phase 4D MRA images. The precision of flow estimation was evaluated using the coefficient of determination (R²) and Bland-Altman analysis. A board-certified neuroradiologist validated the quality of the images and the absence of significant stenosis in the major cerebral arteries.

Results

The generated 4D MRA images closely resembled the ground-truth ASL MRA data, with R² values of 0.92, 0.85, and 0.84 for the internal carotid artery (ICA), proximal middle cerebral artery (MCA), and distal MCA, respectively. Bland-Altman analysis revealed a systematic error of -0.06, with 95% agreement limits ranging from -0.18 to 0.12. Additionally, the model successfully identified flow abnormalities in a subject with left MCA stenosis, displaying a delayed peak and subsequent flattening distal to the stenosis, indicative of reduced blood flow. Visualization of the predicted arterial flow overlaid on the original TOF MRA images highlighted the spatial progression and dynamics of the flow.

Conclusions

The DCNN model effectively generated synthetic 4D MRA images from TOF images, demonstrating its potential to estimate temporal changes in arterial flow accurately. This non-invasive technique offers a promising alternative to conventional methods for visualizing and evaluating healthy and pathological flow dynamics. It has significant potential to improve the diagnosis and treatment of cerebrovascular diseases by providing detailed temporal flow information without the need for contrast agents or invasive procedures. The practical implementation of this model could enable the extraction of dynamic cerebral blood flow information from routine brain MRI examinations, contributing to the early diagnosis and management of cerebrovascular disorders.

## Introduction

Time-of-flight (TOF) magnetic resonance angiography (MRA) has long been a cornerstone in vascular imaging, playing a crucial role in the diagnosis of cerebrovascular disorders and cerebral aneurysms since the early days of clinical MRI [1-6]. Despite emerging advanced technologies, 3D TOF-MRA remains a mainstay for non-contrast vascular imaging in central and peripheral regions due to its ability to provide high spatial resolution images of cerebral arteries (6). This resolution comes from the "inflow effect," one of the three flow-related MR phenomena, in which "fresh" blood entering the imaging slice appears with a high signal [4,7].

Arterial spin labeling (ASL)-MRA, a recent advancement in magnetic resonance angiography, utilizes arterial blood labeled with RF pulses as an endogenous tracer, such as TOF, to represent arterial flow [7-11]. This technique uses short TE settings, which have been reported to mitigate the loss of flow-related signal from laminar and turbulent flows and susceptibility artifacts from metallic devices, thereby improving image quality [12-18].

Furthermore, ASL-MRA allows dynamic signal collection with appropriate timing delays after labeling to achieve 4D MRA with temporal information, which is not possible with TOF and was previously only possible with conventional angiography [14,19-25].

However, when comparing the imaging conditions of ASL-MRA and 3D TOF MRA, it is noteworthy that while ASL-MRA provides temporal information, it does so at the cost of lower spatial resolution and longer imaging times, which are significant disadvantages compared to TOF MRA [18,23].

Although both ASL-MRA and TOF-MRA rely on contrasting arterial flow against the background, the longer signal acquisition time of TOF-MRA, ten times that of ASL in our setting, suggests that it may inherently capture more comprehensive flow information. However, this information is underutilized in conventional 3D TOF-MRA with maximum intensity projection.

Therefore, this study aims to leverage the rich flow information within TOF data using machine learning to extract temporal flow dynamics and generate 4D MRA. Our objective is to develop a method that can accurately estimate changes in arterial flow over time, potentially offering a noninvasive and efficient alternative to existing techniques for healthy subjects and those with pathological flow conditions.

## Materials and methods

Study design

This study aims to generate 4D MRA using machine learning by estimating temporal changes in arterial flow from 3D TOF MRA images. The institutional ethics committee approved the retrospective study with an opt-out option for informed consent.

Data acquisition

All data was collected using a 3T MRI system (Vantage Centurian, Canon Medical Systems) equipped with a 32-channel head coil. The dataset consisted of paired 3D TOF MRA and 4D ASL MRA images from 13 subjects (11 men and 2 women) aged between 42 and 77 years with no history of cerebral vessel occlusion or significant stenosis (Table 1). The imaging parameters for the 3D TOF MRA and 4D ASL MRA are described in Table 2. A board-certified neuroradiologist with more than 28 years of experience confirmed the image quality, the absence of significant stenosis (>50%) in the major cerebral arteries, and flow differences between hemispheres.

**Table 1:** Dataset Characteristics

Parameter	Description
Imaging Modalities	Paired 3D Time-of-Flight (TOF) magnetic resonance angiography (MRA) and 4D Arterial Spin Labeling (ASL) MRA
Number of Subjects	13 subjects
Gender Distribution	11 men, 2 women
Age Range	42 to 77 years
Clinical Background	No history of cerebral vessel occlusion or significant stenosis

**Table 2:** Imaging Parameters of 3D TOF MRA and 4D ASL MRA FOV: Field of View, MRA: Magnetic Resonance Angiography, TOF: Time-of-Flight, ASL: Arterial Spin Labeling, TR: Repetition Time, TE: Echo Time

Parameter	3D TOF MRA	4D ASL MRA
FOV	224mm	200mm
Matrix	224x448	224x224
Slices	152 (1mm thick)	200
TR/TE	18/3.9 msec	3.7/0.1 msec
Flip Angle	15°	6°
Scan Time	2 min 56 sec	11 min 42 sec
Phases	-	8 (150-1550 msec)
Final Voxel Size	512x512x152	256x256x200

Pre-processing

Both 3D TOF and 8-phase ASL MRA images were acquired in DICOM (Digital Imaging and Communications in Medicine) format. The OsiriX DICOM Viewer (Pixmeo SARL, Geneva, Switzerland) was used to resample the 8-phase ASL flow images to match the TOF image positions [26]. The resampled ASL images were exported as 16-bit TIFF images with a window width of 2000 and a window center of 1000, then converted to 8-bit grayscale PNG format and resized to 128x128 pixels using interpolation to reduce data size. The TOF images underwent the same conversion process using the default display settings.

Arterial masks were generated for the TOF and ASL images to focus the model on relevant areas and reduce background noise. Intensity values of the 8-bit grayscale images (0-255) were normalized to the 0-1 range. A 3D binary mask was created by identifying regions within the 8phase ASL images where the difference between the maximum and minimum intensity values exceeded 0.25, representing arterial vessels. Then, this mask was applied to the original ASL images for training.

Data splitting and model validation

The dataset was divided into training and testing sets. Thirteen subjects with various conditions were used for training, while one healthy subject and one post-aneurysm treatment patient were reserved for testing. This ensured an unbiased evaluation of the model's performance. The training set was used to train the model, while the testing set was used to assess its effectiveness in generating 4D MRA images using performance metrics such as accuracy and loss.

Machine learning models

Deep convolutional neural networks (DCNNs) were developed using the Neural Network Console, an open-source Python library (version 3.6.2) provided by Sony Network Communications Inc (Tokyo, Japan) [27].

Two learning processes were implemented:

Modified U-Net

This model was trained to estimate eight-phase flow changes from a single TOF image. It consists of a convolutional layer followed by down-sampling and up-sampling layers, ultimately generating eight normalized flow images [28,29].

Long-Short-Term Memory (LSTM)

To ensure spatial consistency of flow estimation along the z-axis (proximal to distal), the modified Unet was integrated into an LSTM network [30]. This recurrent neural network architecture, specifically designed for time-series data, uses the modified U-Net as its memory cell. It employs four-layer convolutional neural networks for the forget input and output gates. By processing 152 consecutive axial images, the LSTM maintains long-term memory along the z-axis, preserving the estimated flow's spatial consistency in axial and planar dimensions.

**Figure 1:**
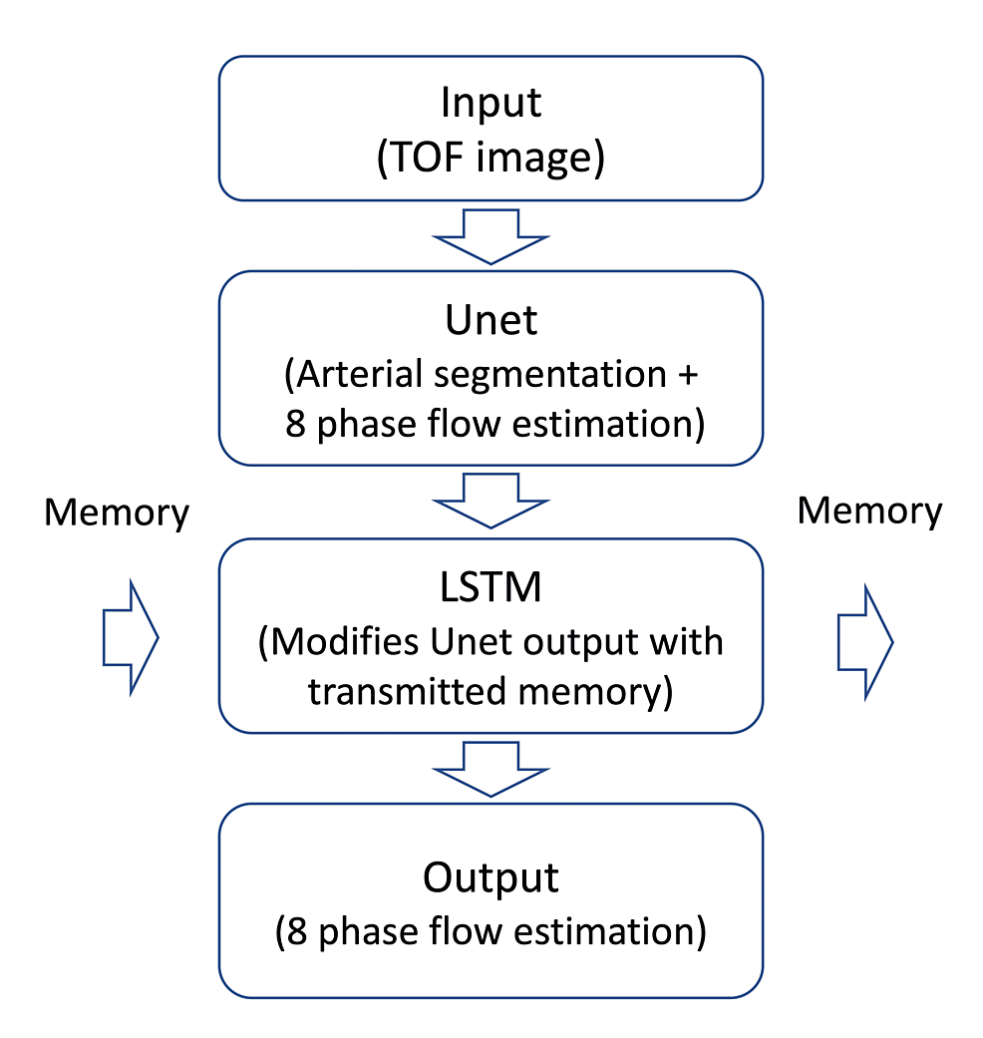
Structure of the machine learning model synthetic 8-phase flow from the TOF signal The modified Unet extracts arterial regions from the time-of-flight (TOF) image and converts the intravascular signal into an 8-phase flow. The long-short-term memory (LSTM) model was used to maintain the spatial consistency between the transformation from TOF to eight-phase flow images and the direction of arterial flow in the brain. The detailed structure of these models can be seen in the Appendices.

The architecture of the combined model is illustrated in Figure 1 and is further detailed in the supplementary materials.

Training

The model used the mean squared error as the loss function, measuring the pixel-level discrepancy between the predicted and ground-truth normalized eight-phase ASL flow signals. Training parameters included a maximum of 1000 epochs, an optimization algorithm of Adam with an initial learning rate of 0.001, α and β values of 0.001 and 0.9 respectively, and a learning rate scheduler of cosine annealing.

Evaluation

Two metrics were used to evaluate the model's performance: the coefficient of determination (R²), which assesses the goodness of fit between the predicted 8-phase flow and the actual ASL flow signal, and Bland-Altman analysis, which evaluates the agreement between the predicted and actual flow by calculating the systematic error (mean difference), precision (standard deviation), and 95% agreement limits. The regions of interest were manually placed in the internal carotid artery and the proximal and distal middle cerebral arteries to analyze the relationship between predicted and actual flow signals.

## Results

**Figure 2:**
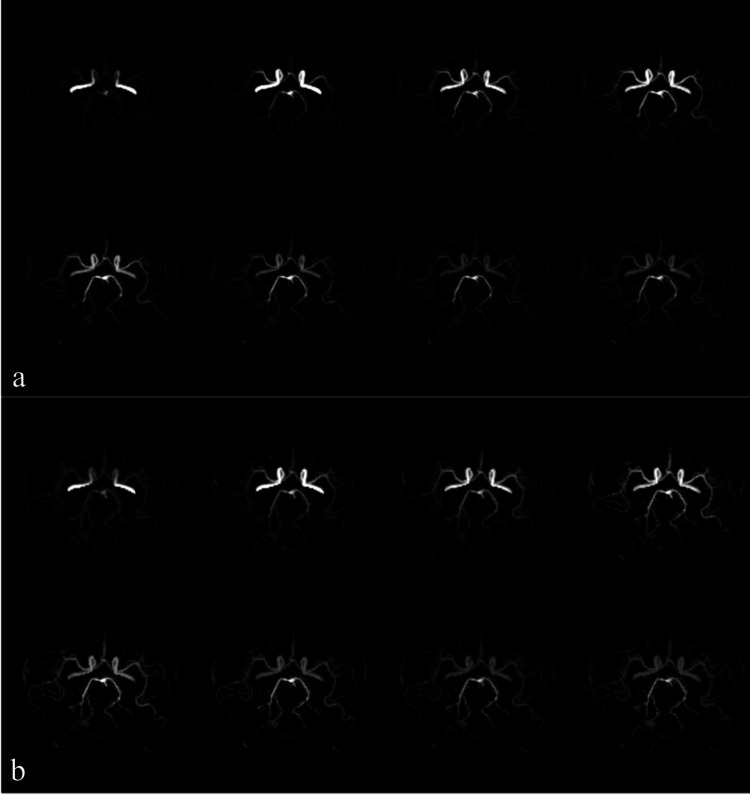
Comparison of synthetic and ground-truth 4D MRA. (a) Synthetic 8-phase 4D magnetic resonance angiography (MRA) generated from time-of-flight (TOF) data using the deep convolutional neural network (DCNN) model. (b) 4D ground truth MRA acquired by arterial spin labeling (ASL). Both images are shown in the same axial projection.

**Figure 3:**
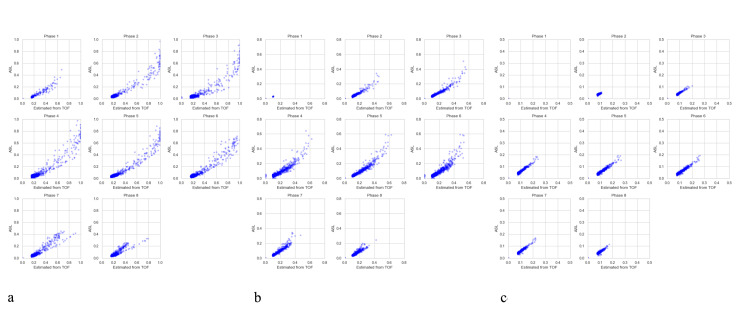
Scatter plots comparing the estimated 8-phase flow from TOF with the actual ASL flow in a healthy test subject. (a) Internal carotid artery (ICA). (b) Proximal middle cerebral artery (MCA). (c) Distal MCA. The x-axis represents the estimated flow from time-of-flight (TOF), and the y-axis represents the corresponding arterial spin labeling (ASL) signal value, with both scales normalized between 0 and 1.

**Figure 4:**
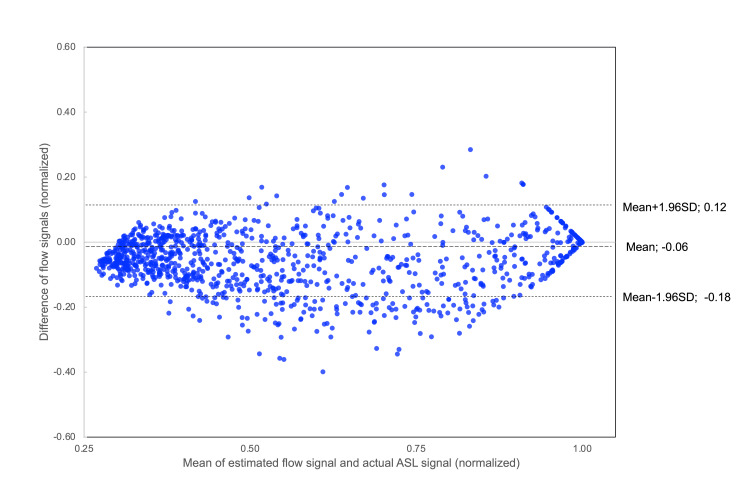
Bland-Altman graph comparing estimated 8-phase flow with actual arterial spin labeling (ASL) flow in the internal carotid artery. The x-axis represents the mean difference between the two signals, and the y-axis represents the mean of the two signals. The systematic error (mean difference) is -0.06, and the 95% limits of agreement are 0.12 and -0.18, indicating good agreement between the estimated and actual flow signals.

The DCNN model successfully generated 8-phase 4D MRA images that resemble the reference ASL MRA data (Figure 2). In a healthy test subject, the R² values for flow estimation were 0.92, 0.85, and 0.84 for the internal carotid artery, the proximal middle cerebral artery (MCA) and the distal MCA, respectively (Figure 3). These findings were statistically significant (p < 0.001). Bland-Altman analysis showed a systematic error of -0.06 with 95% agreement limits ranging from -0.18 to 0.12 (Figure 4).

**Figure 5:**
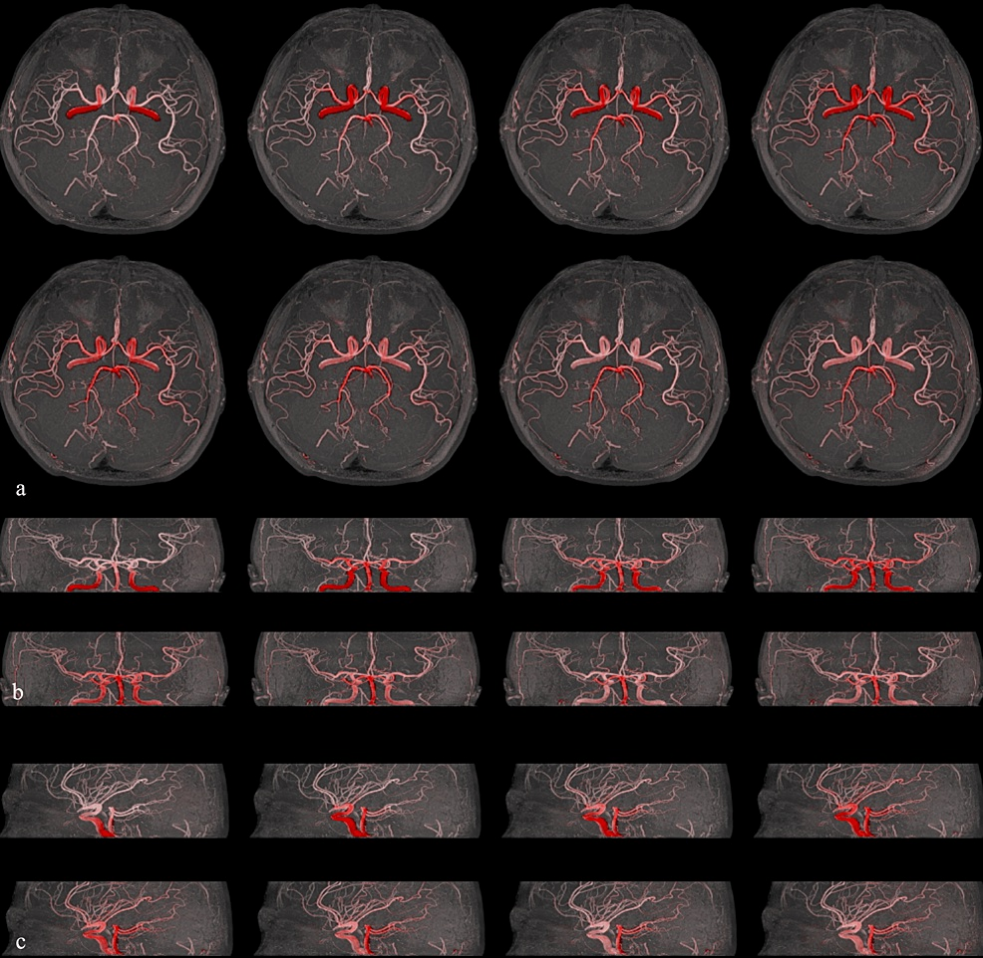
Visualization of synthetic 8-phase flow progression overlayed on the original 3D TOF MRA. The estimated flow is depicted in red, and the original time-of-flight (TOF) magnetic resonance angiography (MRA) is superimposed in white. (a) Axial projection. (b) Lateral projection. This visualization allows for the observation of vascular structures and the progression of arterial flow over time.

**Figure 6:**
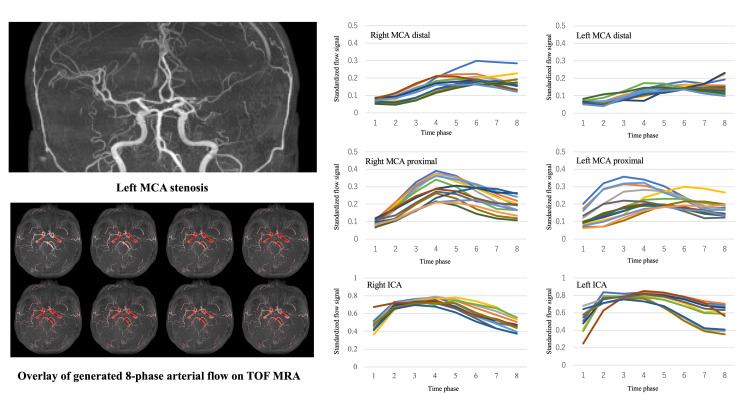
Synthetic 4D MRA in a left middle cerebral artery stenosis case. The top left is a frontal view of time-of-flight (TOF) magnetic resonance angiography (MRA), and the bottom left is an overlay of the generated 8-phase arterial signal to TOF MRA. On the right are the time signal changes of the generated 8-phase flow signals of the left and right internal carotid arteries (ICAs) and the proximal and distal middle cerebral arteries (MCAs). Generated time signal curve showing a delayed peak distal to the stenosis and subsequent flattening, indicating reduced blood flow.

Overlaying the predicted arterial flow (in red) on the original 3D TOF MRA image visualizes the flow's reach throughout the vasculature (Figure 5). In a test subject with left MCA stenosis, the 8-phase flow signal generated exhibited a delayed peak and subsequent flattening distal to the stenosis, indicating reduced flow (Figure 6).

## Discussion

This study demonstrates the feasibility of using a DCNN model to generate synthetic 4D MRA from 3D TOF images. The model effectively estimated temporal changes in arterial flow in healthy subjects, achieving high R² values and good agreement with ground-truth ASL MRA data. Furthermore, visualization of flow progression and identification of flow abnormalities in a stenosis case highlight the potential of this technique for clinical applications.

Applications and unmet needs of current TOF MRA

TOF-MRA provides static information about the structure of arteries through MIP processing, similar to traditional cerebral angiography. However, it does not provide dynamic information about the contrast agent's circulation dynamics, as this information is lost during MIP processing. Considering the principles of TOF, it can be hypothesized that dynamic information about arterial blood flow is also latent within TOF signals.

ASL is a technique that can extract dynamic information from arteries, but compared to TOF-MRA, ASL-MRA has the disadvantages of longer imaging times and lower spatial resolution. The main aim of this study is to extend the utility of TOF-MRA by using a machine learning model trained on paired TOF and ASL data to extract the latent dynamic information from TOF signals.

In general, brain MRI examinations use TOF signals to indicate the static information of arteries, showing their shape. This has proven effective in detecting aneurysms and vascular stenosis. Given the principles of TOF signals, it can be inferred that dynamic information about arteries is also latent.

Expected clinical impact of 4D MRA

This proposed method offers several practical applications that could benefit general clinical practice:

Extending the Utility of TOF MRA: The proposed method enhances the capabilities of TOF MRA beyond its traditional use by extracting dynamic flow information from TOF signals.

Utilizing Dynamic Information: The dynamic information lost during MIP processing can provide a more comprehensive view of arterial flow.

Screening for Cerebrovascular Disorders: During routine screenings, the proposed method could detect minor blood flow reductions from time-series blood flow changes estimated from TOF, potentially predicting the risk of cerebrovascular disorders.

Comparison of TOF and ASL techniques

TOF and ASL MRA exploit the contrast between flowing blood and static tissue to visualize vascular structures. However, they differ in their signal acquisition time and the type of information they provide. ASL, with its rapid filling of the k-space and short echo time, achieves a temporal resolution of 200 milliseconds, enabling the acquisition of multiple 3D images within a short timeframe. In contrast, TOF MRA has a longer acquisition time, approximately ten times longer than ASL in our study. This difference inspired the current research, as the longer acquisition time of TOF may inherently capture more comprehensive flow information compared to the snapshot-like nature of ASL.

Model architecture and rationale

The combination of U-Net and LSTM networks was chosen for this study due to their complementary strengths. The U-Net architecture excels at image segmentation and transformation tasks, allowing for the extraction of arterial regions from TOF images and estimating eight-phase flow changes. The LSTM network, with its ability to retain long-term memory, ensures spatial consistency of flow estimation along the z-axis, providing a more accurate representation of the flow dynamics within the vasculature.

Limitations

This preliminary study has several limitations. To achieve feasible training times, the spatial resolution of the output images was reduced compared to the original TOF MRA. Furthermore, the training dataset was limited in size and lacked sufficient pathological flow data. The preprocessing steps involved converting DICOM data to lower-quality formats and using interpolation, which may have resulted in some loss of the original MR signal information. Although these steps were necessary due to current limitations in data handling capacity, they may affect the authenticity of the images. Future studies should incorporate larger and more diverse datasets, including various pathologies, to further validate and improve the generalizability of the model. Additionally, as processing environments advance, it will be possible to handle higher-quality data, thereby reducing the need for such preprocessing steps and preserving the integrity of the original MR signals.

Future directions

Future research can be extended in several ways. Improving the spatial resolution may involve exploring alternative network architectures or employing data-augmentation techniques, which could enable the generation of high-resolution 4D MRA while maintaining efficient training times. Expanding the training dataset to include a wider range of pathologies and flow conditions will enhance the model’s generalizability and robustness. Additionally, implementing methods to quantify the uncertainty associated with the model's predictions can improve its reliability for clinical decision-making. Finally, investigating techniques to understand how the model arrives at its predictions will increase trust and acceptance in clinical settings.

## Conclusions

This study demonstrates the potential of deep learning techniques to extract temporal flow information from TOF MRA and generate synthetic 4D MRA. The proposed method offers a promising noninvasive approach to visualizing and evaluating arterial flow dynamics, which may aid in the diagnosis and management of cerebrovascular diseases.

## Figures and Tables

**Figure 7:**
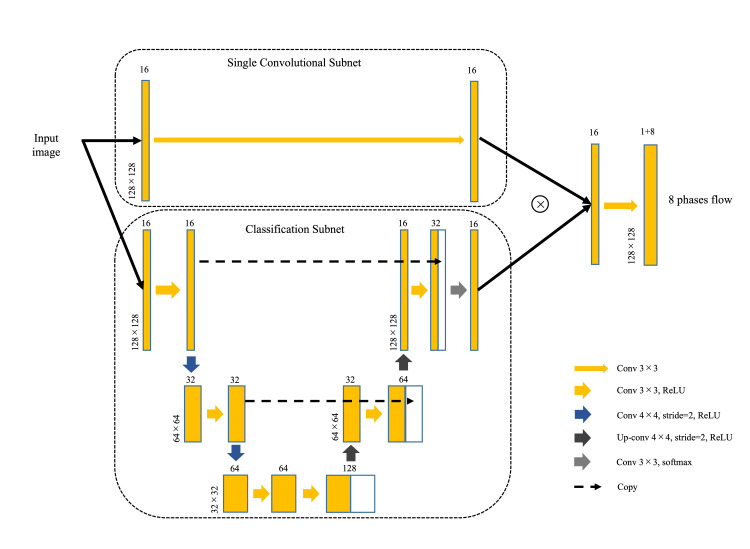
Detailed architecture of the modified Unet network used to estimate the 8-phase flow from the time-of-flight (TOF) signal. The network uses a combination of convolutional layers and rectified linear units (ReLU) to extract features and generate flow estimations.

**Figure 8:**
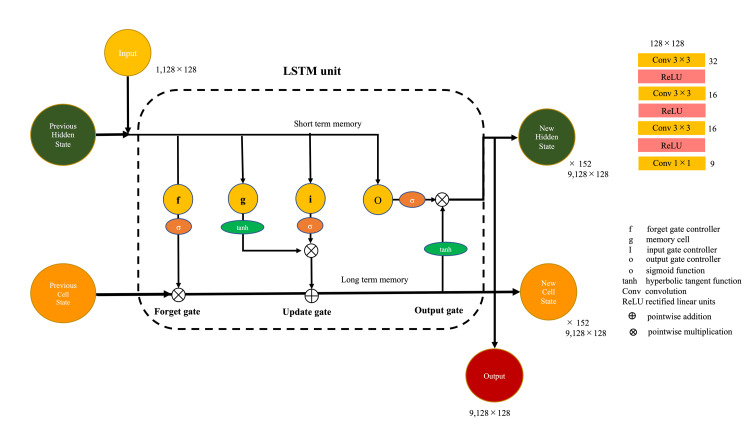
Detailed architecture of the long-short-term memory (LSTM) network used to maintain spatial consistency of flow estimation along the z-axis. The LSTM employs memory cells and gate controllers to regulate the information flow and preserve temporal relationships within the generated 4D magnetic resonance angiography (MRA).
